# *In vitro* activity of aztreonam–nacubactam and cefepime–nacubactam against a global collection of clinically important Gram-negative Bacilli collected in 2021–2023

**DOI:** 10.1093/jacamr/dlag061

**Published:** 2026-05-05

**Authors:** James A Karlowsky, Mark Estabrook, Eisuke Suzuki, Atsuyuki Shimizu, Hayato Okade

**Affiliations:** IHMA, Schaumburg, IL 60173, USA; Department of Medical Microbiology and Infectious Diseases, Max Rady College of Medicine, University of Manitoba, Winnipeg, MB R3E 0J9, Canada; IHMA, Schaumburg, IL 60173, USA; R&D Division, Meiji Seika Pharma Co. Ltd., Tokyo, Japan; R&D Division, Meiji Seika Pharma Co. Ltd., Tokyo, Japan; R&D Division, Meiji Seika Pharma Co. Ltd., Tokyo, Japan

## Abstract

**Objectives:**

We assessed the *in vitro* activity of aztreonam–nacubactam and cefepime–nacubactam against a 2021–2023 global collection of clinical isolates of Gram-negative bacilli (12 613 *Enterobacterales*, 3748 *Pseudomonas aeruginosa* and 601 *Acinetobacter baumannii*). Nacubactam is a novel diazabicyclooctane β-lactamase inhibitor that also possesses intrinsic antimicrobial (PBP2 inhibition) and β-lactam enhancer (for β-lactams that bind PBP3 and other non-PBP2 PBPs) activities against *Enterobacterales*. Aztreonam–nacubactam and cefepime–nacubactam are in clinical development for the treatment of complicated urinary tract infections, acute uncomplicated pyelonephritis and various infections caused by carbapenem-resistant *Enterobacterales* (CRE).

**Methods:**

MICs were determined for aztreonam–nacubactam (1:1 ratio), cefepime–nacubactam (1:1 ratio) and 13 comparators using the CLSI broth microdilution method.

**Results:**

For all *Enterobacterales* isolates, the MIC_90_s of aztreonam–nacubactam and cefepime–nacubactam were 1 and 0.5 mg/L, respectively; 99.8% of all *Enterobacterales* isolates and 99.8% of CRE isolates (*n* = 917) were inhibited by aztreonam–nacubactam at 4 mg/L and by cefepime–nacubactam at 8 mg/L. Both aztreonam-resistant and cefepime-resistant isolate MICs demonstrated a modal decrease of 64-fold by the addition of nacubactam at a 1:1 ratio. Aztreonam–nacubactam and cefepime–nacubactam were active against the majority of *Enterobacterales* isolates that were resistant to aztreonam–avibactam, ceftazidime–avibactam, imipenem–relebactam, meropenem–vaborbactam and cefiderocol. The MIC_90_s for aztreonam–nacubactam and cefepime–nacubactam were 16 and 8 mg/L for *P. aeruginosa* and >64 and 64 mg/L for *A. baumannii*.

**Discussion:**

The results of this study support the continued development of aztreonam–nacubactam and cefepime–nacubactam as potential therapies for infections caused by CRE where resistance or potential toxicity to marketed antimicrobial agents excludes their use.

## Introduction

Infections attributable to carbapenem-resistant *Enterobacterales* (CRE) are growing in prevalence worldwide and are associated with greater patient morbidity and mortality, longer hospital stays and increased medical costs compared with carbapenem-susceptible infections.^1–5^ The mechanisms of resistance in CRE frequently include β-lactamases and isolates often have MDR phenotypes, leaving care providers with few treatment options.^1^ The WHO has identified CRE as a critical priority bacterial pathogen for research and development of new antimicrobial agents.^5^ β-lactams are generally preferred to other treatment options (e.g. fluoroquinolones) because they possess better side effect profiles.

Newer, marketed β-lactam/β-lactamase inhibitor combinations include either a diazabicyclooctane (DBO; aztreonam–avibactam, ceftazidime–avibactam, imipenem–relebactam) or bicyclic boronate (meropenem–vaborbactam) inhibitor that is active against Gram-negative bacilli carrying Ambler class A serine-based carbapenemases (e.g. KPC), acquired class C (AmpC) β-lactamases (e.g. CMY, DHA) and some class D enzymes (e.g. OXA-48-like). Avibactam, relebactam and vaborbactam do not inhibit Ambler class B metallo-β-lactamases (MBLs). Aztreonam–avibactam and cefiderocol have both demonstrated *in vitro* against MBL-producing Gram-negative bacilli and may provide clinical benefit to patients infected with susceptible isolates.^1,2^

Nacubactam is a novel DBO β-lactamase inhibitor currently under development by Meiji Seika Pharma Co., Ltd. (Tokyo, Japan) as a stand-alone drug to be co-administered with aztreonam or cefepime in contrast to other β-lactam/β-lactamase inhibitor combinations which have been developed only in combination with a particular partner. Its mechanism of action involves three distinct processes.^6–9^ First, nacubactam inhibits class A, class C and some class D serine β-lactamases (including ESBLs, KPCs and OXA-48-like β-lactamases) by covalently binding to their active sites. Second, nacubactam directly inhibits penicillin-binding protein 2 (PBP2) in most clinically relevant *Enterobacterales* species, converting bacilli into spherical forms. Third, by binding to PBP2, nacubactam enhances the activities of β-lactams that bind PBP3 (e.g. cephalosporins, carbapenems and monobactams) and other non-PBP2 PBPs. A Phase III noninferiority clinical trial comparing the efficacy and safety of aztreonam or cefepime in combination with nacubactam to imipenem/cilastatin in patients with complicated urinary tract infections or acute pyelonephritis was successfully completed in March 2025 (Integral-1 trial, jRCT2031230075, NCT05887908).^10^ A second Phase III clinical trial (Integral-2 study, jRCY2031230076, NCT05905055) assessing the efficacy and safety of aztreonam–nacubactam and cefepime–nacubactam versus the best available therapy for patients with CRE infections was also successfully completed (data release pending).^10^

Currently, published *in vitro* antimicrobial susceptibility data for aztreonam–nacubactam and cefepime–nacubactam tested against collections of clinical isolates are limited.^8,11–13^ This *in vitro* surveillance study of 16 962 global clinical isolates of Gram-negative bacilli was conducted to supplement earlier descriptive studies testing limited numbers of clinical isolates and laboratory strains and clinical trial antimicrobial susceptibility testing data.

## Methods

### Isolate collection

The 16 962 clinical isolates of Gram-negative bacilli included in this study were selected from 3 years (2021, 2022 and 2023) of frozen, stocked global surveillance study isolates maintained by IHMA (Schaumburg, IL, USA). The isolates were collected by 93 clinical laboratories in 33 countries (Table S1, available as Supplementary data at *JAC-AMR* Online). Isolate infection source and year of collection data are provided in Table S2. The identities of all isolates were confirmed by IHMA using MALDI-TOF mass spectrometry (Bruker Daltonics, Billerica, MA, USA).

### Antimicrobial susceptibility testing

Nacubactam (lot number BS1807SB03) was provided by Meiji Seika Pharma Co., Ltd. All other antimicrobial powders were purchased from United States Pharmacopeia (Rockville, MD), MedChemExpress (Monmouth Junction, NJ), Sigma-Aldrich, Inc. (St. Louis, MO), Biochempartner (Shanghai, China) and Selleckchem (Houston, TX). All antimicrobial agents were dissolved and diluted following CLSI instructions.^14^ Broth microdilution panels were prepared with cation-adjusted Mueller–Hinton broth (Sensititre, Thermo Scientific, Waltham, MA) following reference CLSI methodology.^14,15^ Panels were frozen at −80°C and thawed to room temperature prior to use. Doubling dilutions for aztreonam–nacubactam and cefepime–nacubactam were prepared at a 1:1 ratio of the two components.^14^ One-to-one ratios of aztreonam:nacubactam and cefepime:nacubactam are recommended by CLSI for testing these combinations because both agents have antibacterial activity, and the 1:1 ratio eliminates activity bias due to either of the components. Other combination agents were each tested at a fixed β-lactamase inhibitor concentration of 4 mg/L (aztreonam–avibactam, ceftazidime–avibactam, imipenem–relebactam and piperacillin–tazobactam) or 8 mg/L (meropenem–vaborbactam) in combination with doubling dilutions of the companion β-lactam.^14^ Iron-depleted cation-adjusted Mueller–Hinton broth was prepared following the method described by CLSI^14^ and used to test cefiderocol. CLSI recommended quality control testing was performed each day of testing.^14^

Broth microdilution MIC endpoints were read following panel incubation at 35°C for 16–20 h in ambient air.^14^ MICs were interpreted using both CLSI^14^ and EUCAST breakpoints.^16^ CRE isolates were defined by resistance (MIC ≥4 mg/L) to either imipenem or meropenem.^14^

## Results

For all isolates of *Enterobacterales* and in each year from 2021 to 2023, the aztreonam–nacubactam MIC_90_ was 1 mg/L (Table 1). The overall (2021–2023) MIC_90_ for each individual species of *Enterobacterales* was also ≤1 mg/L, ranging from an MIC_90_ of ≤0.03 mg/L for *Proteus* species other than *Proteus mirabilis* to 1 mg/L for *Citrobacter freundii* complex, *Enterobacter cloacae* complex, *Klebsiella aerogenes*, *Klebsiella pneumoniae* and *Providencia* species. Aztreonam–nacubactam was less active against *P. aeruginosa* (MIC_90_, 16 mg/L) and *Acinetobacter baumannii* (MIC_90_, >64 mg/L) than against *Enterobacterales*.

**Table 1.** *In vitro* activity of aztreonam–nacubactam against 16 962 clinical isolates of Gram-negative bacilli tested in three consecutive annual studies

	2021				2022				2023				All			
		mg/L				mg/L				mg/L				mg/L		
Organism	*n*	MIC range	MIC_50_	MIC_90_	*n*	MIC range	MIC_50_	MIC_90_	*n*	MIC range	MIC_50_	MIC_90_	*n*	MIC range	MIC_50_	MIC_90_
*Enterobacterales*	3656	≤0.03–16	0.06	1	3695	≤0.03–4	0.06	1	5262	≤0.03–>64	0.06	1	12 613	≤0.03–>64	0.06	1
*Citrobacter freundii* complex	77	≤0.03–4	0.12	1	69	≤0.03–1	0.12	1	119	≤0.03–2	0.12	0.5	265	≤0.03–4	0.12	1
*Citrobacter koseri*	67	≤0.03–1	0.06	0.12	64	≤0.03–1	0.06	0.12	83	≤0.03–2	0.06	0.12	214	≤0.03–2	0.06	0.12
*Enterobacter cloacae* complex	305	≤0.03–2	0.12	1	314	≤0.03–4	0.12	1	468	≤0.03–4	0.12	1	1087	≤0.03–4	0.12	1
*Enterobacter bugandensis*	49	≤0.03–2	0.06	1	55	≤0.03–1	0.06	0.5	58	≤0.03–2	0.06	0.5	162	≤0.03–2	0.06	0.5
*Escherichia coli*	1297	≤0.03–8	0.12	0.5	1301	≤0.03–4	0.06	0.5	1886	≤0.03–>64	0.06	0.5	4484	≤0.03–>64	0.06	0.5
*Klebsiella aerogenes*	108	≤0.03–2	0.12	1	71	≤0.03–4	0.25	1	60	≤0.03–2	0.06	1	239	≤0.03–4	0.12	1
*Klebsiella oxytoca*	3	≤0.03–0.5	ND^a^	ND	79	≤0.03–1	0.06	0.25	103	≤0.03–4	0.06	0.5	185	≤0.03–4	0.06	0.5
*Klebsiella pneumoniae*	1132	≤0.03–4	0.12	1	1138	≤0.03–4	0.06	1	1623	≤0.03–>64	0.06	1	3893	≤0.03–>64	0.06	1
*Klebsiella variicola*	40	≤0.03–2	0.06	0.25	29	≤0.03–0.25	0.06	0.25	38	≤0.03–1	≤0.03	0.25	107	≤0.03–2	0.06	0.25
*Morganella morganii*	80	≤0.03–8	≤0.03	0.25	96	≤0.03–0.5	≤0.03	0.25	115	≤0.03–2	≤0.03	0.06	291	≤0.03–8	≤0.03	0.25
*Proteus mirabilis*	265	≤0.03–16	≤0.03	0.12	260	≤0.03–4	≤0.03	0.12	354	≤0.03–>64	≤0.03	0.12	879	≤0.03–>64	≤0.03	0.12
Other *Proteus* spp.	38	≤0.03	≤0.03	≤0.03	13	≤0.03–0.06	≤0.03	≤0.03	42	≤0.03–1	≤0.03	0.06	93	≤0.03–1	≤0.03	≤0.03
*Providencia* spp.	14	≤0.03–2	≤0.03	1	47	≤0.03–4	0.06	2	32	≤0.03–4	≤0.03	0.12	93	≤0.03–4	≤0.03	1
*Serratia marcescens*	126	0.06–4	0.12	0.5	148	0.06–4	0.12	0.25	160	≤0.03–>64	0.12	1	434	≤0.03–>64	0.12	0.5
Other	55	≤0.03–2	0.12	0.5	11	≤0.03–1	0.12	1	121	≤0.03–2	0.12	0.5	187	≤0.03–2	0.12	0.5
*Pseudomonas aeruginosa*	1114	≤0.03–>64	4	16	1083	≤0.03–>64	4	16	1551	≤0.03–>64	4	16	3748	≤0.03–>64	4	16
*Acinetobacter baumannii*	196	1–>64	32	>64	188	0.5–>64	32	>64	217	2–>64	32	>64	601	0.5–>64	32	>64

^a^ND, not determined.

For all isolates of *Enterobacterales*, and in each year from 2021 to 2023, the cefepime–nacubactam MIC_90_ was 0.5 mg/L (Table 2). The overall MIC_90_ for each individual species of *Enterobacterales* was also ≤0.5 mg/L for all species except *E. coli* (MIC_90_, 1 mg/L) and *Providencia* species (MIC_90_, 4 mg/L). Cefepime–nacubactam was less active against *P. aeruginosa* (MIC_90_, 8 mg/L) and *A. baumannii* (MIC_90_, 64 mg/L) than against *Enterobacterales*.

**Table 2.** *In vitro* activity of cefepime-nacubactam against 16 962 clinical isolates of Gram-negative bacilli from three consecutive annual studies

	2021				2022				2023				All			
		mg/L				mg/L				mg/L				mg/L		
Organism	*n*	MIC range	MIC_50_	MIC_90_	*n*	MIC range	MIC_50_	MIC_90_	*n*	MIC range	MIC_50_	MIC_90_	*n*	MIC range	MIC_50_	MIC_90_
*Enterobacterales*	3656	≤0.008–64	0.06	0.5	3695	≤0.008–>64	0.06	0.5	5262	≤0.008–64	0.06	0.5	12 613	≤0.008–>64	0.06	0.5
*Citrobacter freundii* complex	77	0.015–32	0.03	0.5	69	0.015–4	0.06	0.5	119	0.015–2	0.03	0.25	265	0.015–32	0.03	0.25
*Citrobacter koseri*	67	≤0.008–1	0.03	0.12	64	0.015–1	0.03	0.12	83	0.015–1	0.03	0.12	214	≤0.008–1	0.03	0.12
*Enterobacter cloacae* complex	305	≤0.008–4	0.06	0.5	314	0.015–4	0.06	0.5	468	≤0.008–4	0.06	0.5	1087	≤0.008–4	0.06	0.5
*Enterobacter bugandensis*	49	0.015–0.25	0.06	0.12	55	0.015–0.25	0.06	0.25	58	0.015–0.5	0.06	0.12	162	0.015–0.5	0.06	0.12
*Escherichia coli*	1297	≤0.008–8	0.06	0.5	1301	≤0.008–4	0.06	0.25	1886	≤0.008–32	0.06	0.5	4484	≤0.008–32	0.06	0.5
*Klebsiella aerogenes*	108	0.03–4	0.06	0.25	71	0.03–8	0.06	0.5	60	0.015–2	0.06	0.25	239	0.015–8	0.06	0.25
*Klebsiella oxytoca*	3	0.03–0.12	ND^a^	ND	79	0.015–2	0.03	0.12	103	0.015–4	0.03	0.25	185	0.015–4	0.03	0.12
*Klebsiella pneumoniae*	1132	0.015–64	0.06	1	1138	≤0.008–64	0.06	2	1623	≤0.008–64	0.06	1	3893	≤0.008–64	0.06	1
*Klebsiella variicola*	40	0.015–1	0.03	0.25	29	0.015–0.25	0.03	0.12	38	0.015–0.5	0.03	0.12	107	0.015–1	0.03	0.12
*Morganella morganii*	80	≤0.008–8	0.03	0.12	96	0.015–0.25	0.03	0.06	115	≤0.008–1	0.03	0.06	291	≤0.008–8	0.03	0.06
*Proteus mirabilis*	265	0.015–64	0.06	0.25	260	0.015–8	0.06	0.25	354	≤0.008–32	0.06	0.25	879	≤0.008–64	0.06	0.25
Other *Proteus* spp.	38	0.015–0.12	0.06	0.06	13	0.03–0.06	0.06	0.06	42	0.03–1	0.06	0.12	93	0.015–1	0.06	0.12
*Providencia* spp.	14	0.015–16	0.015	4	47	0.015–>64	0.12	64	32	0.015–8	0.03	0.06	93	0.015–>64	0.06	4
*Serratia marcescens*	126	0.03–16	0.12	0.25	148	0.03–2	0.06	0.25	160	0.03–8	0.06	0.5	434	0.03–16	0.12	0.25
Other	55	0.06–8	0.06	0.25	11	0.03–1	0.06	0.5	121	≤0.008–2	0.06	0.5	187	≤0.008–8	0.06	0.5
*Pseudomonas aeruginosa*	1114	0.03–>64	2	8	1083	0.06–>64	2	8	1551	0.03–>64	2	8	3748	0.03–>64	2	8
*Acinetobacter baumannii*	196	0.25–>64	16	32	188	0.5–>64	16	64	217	0.5–>64	16	64	601	0.25–>64	16	64

^a^ND, not determined.

**Figure 1.**
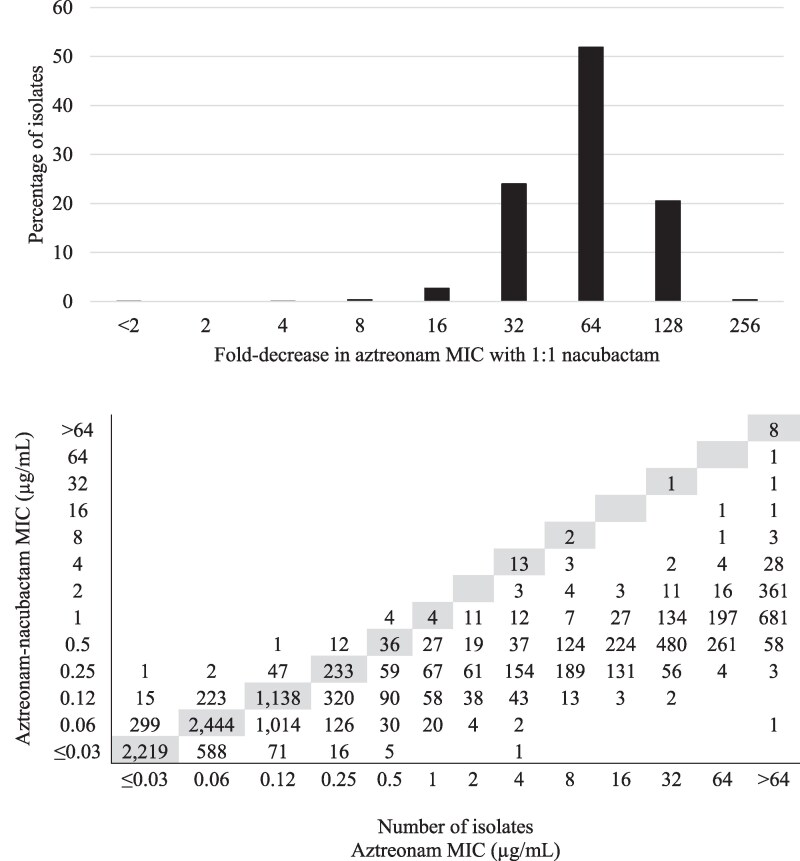
*Top*: fold-decrease in aztreonam MIC with the addition of nacubactam at a 1:1 ratio against 1558 *Enterobacterales* isolates that tested resistant to aztreonam by CLSI breakpoints (≥16 mg/L). This figure only includes isolates that tested with on-scale MIC values for both aztreonam and aztreonam–nacubactam (i.e. between 0.06 and 64 mg/L for both aztreonam and the aztreonam–nacubactam combination). Using only on-scale MICs led to the exclusion of 1146 isolates testing with aztreonam MICs of >64 mg/L. *Bottom*: per-isolate comparison of the aztreonam–nacubactam MIC and the aztreonam MIC for all 12 613 isolates of *Enterobacterales* tested. Shaded boxes denote identical aztreonam and aztreonam–avibactam MICs.

**Figure 2.**
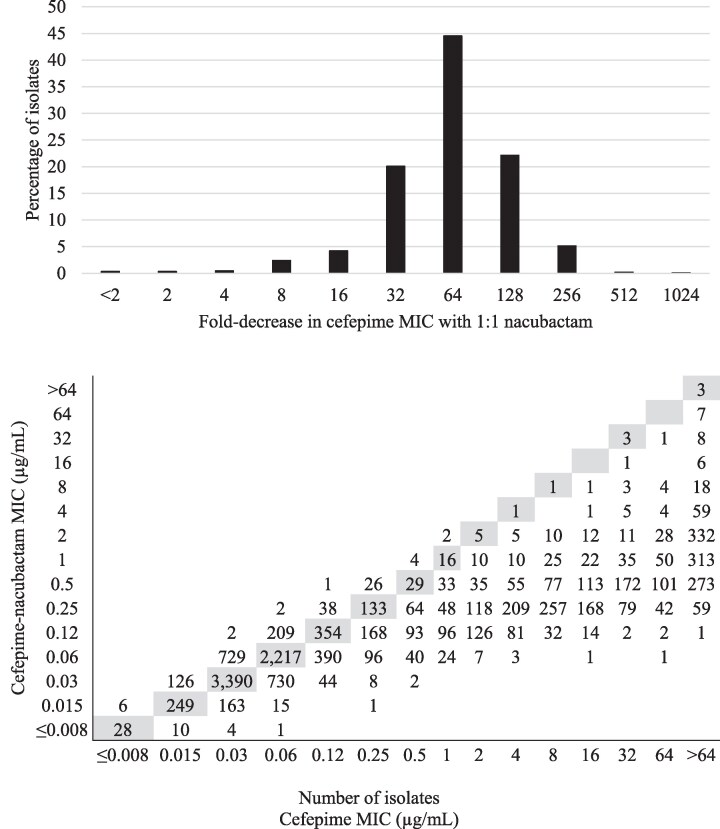
*Top*: fold decrease in cefepime MIC with the addition of nacubactam at a 1:1 ratio against 876 *Enterobacterales* isolates that tested resistant to cefepime by CLSI breakpoints (≥16 mg/L). This figure only includes isolates that tested with on-scale MIC values for both cefepime and cefepime–nacubactam (i.e. between 0.015 and 64 mg/L for both cefepime and the cefepime–nacubactam combination). Using only on-scale MICs led to the exclusion of 1079 isolates testing with cefepime MICs of >64 mg/L *Bottom*: per-isolate comparison of the cefepime–nacubactam MIC and the cefepime MIC for all 12 613 isolates of *Enterobacterales* tested. Shaded boxes denote identical cefepime and cefepime-avibactam MICs.

The MIC_90_ values for aztreonam, cefepime and nacubactam were each 64 mg/L compared to 1 mg/L for aztreonam–nacubactam and 0.5 mg/L for cefepime–nacubactam against the 12 613 clinical isolates of *Enterobacterales* tested (Table 3). Aztreonam-resistant isolates of *Enterobacterales* (MIC, ≥16 mg/L) testing with on-scale MIC values for both aztreonam and aztreonam–nacubactam showed a modal decrease of 64-fold in aztreonam MIC by the addition of nacubactam at a 1:1 ratio (Figure 1). Cefepime-resistant isolates (MIC, ≥16 mg/L) testing with on-scale MIC values for both cefepime and cefepime–nacubactam showed a modal decrease of 64-fold in cefepime MICs by the addition of nacubactam at a 1:1 ratio (Figure 2). Percent susceptible values were 99.7% for aztreonam–avibactam, 99.3% (CLSI) and 97.3% (EUCAST) for cefiderocol, 97.0% for ceftazidime–avibactam, 96.1% (CLSI) and 96.4% (EUCAST) for meropenem–vaborbactam and 93.5% (CLSI) and 95.7% (EUCAST) for imipenem–relebactam. Imipenem-resistant isolates accounted for 5.2% (EUCAST) to 7.0% (CLSI) of *Enterobacterales* isolates tested, and meropenem-resistant isolates accounted for 4.8% (EUCAST) to 5.8% (CLSI) of isolates. Comparative agent data for isolates of *P. aeruginosa* and *A. baumannii* are provided in Tables S3 and S4, respectively.

**Table 3.** *In vitro* activity of aztreonam–nacubactam, cefepime–nacubactam and comparator agents against 12 613 clinical isolates of *Enterobacterales*

				MIC interpretation^a^					
	mg/L			CLSI				EUCAST	
Antibacterial agent	MIC range	MIC_50_	MIC_90_	% S	% SDD	% I	% R	% S	% R
Aztreonam	≤0.03–>64	0.12	64	75.9	NA	2.7	21.4	72.7	24.1
Aztreonam–avibactam	≤0.015–>64	0.03	0.12	99.7	NA	0.2	0.1	99.7	0.3
Aztreonam–nacubactam	≤0.03–>64	0.06	1	NA	NA	NA	NA	NA	NA
Cefepime	≤0.008–>64	0.06	64	78.4	6.1	NA	15.5	76.0	18.7
Cefepime–nacubactam	≤0.008–>64	0.06	0.5	NA	NA	NA	NA	NA	NA
Cefiderocol	≤0.03–>32	0.25	1	99.3	NA	0.4	0.3	97.3	2.7
Ceftazidime	≤0.03–>32	0.25	>32	75.8	NA	2.9	21.3	71.5	24.2
Ceftazidime–avibactam	≤0.015–>64	0.12	0.5	97.0	NA	0	3.0	97.0	3.0
Colistin	≤0.12–>16	0.25	>16	0	NA	82.0	18.0	82.0	18.0
Imipenem	≤0.03–>32	0.25	2	89.7	NA	3.3	7.0	93.0	5.2
Imipenem–relebactam	≤0.03–>32	0.12	0.5	93.5	NA	2.1	4.3	95.7	4.3
Meropenem	≤0.004–>16	0.03	0.12	93.8	NA	0.4	5.8	94.2	4.8
Meropenem–vaborbactam	≤0.004–>16	0.03	0.06	96.1	NA	0.4	3.6	96.4	3.6
Nacubactam	≤0.25–>256	2	64	NA	NA	NA	NA	NA	NA
Piperacillin–tazobactam	≤0.25–>32	2	>32	81.0	4.0	NA	15.1	81.0	19.0

^a^S, susceptible; SDD, susceptible-dose dependent; I, intermediate; R, resistant; NA, there are no MIC breakpoints available for this agent, or there are no MIC breakpoint criteria for this interpretative category, or the MIC breakpoint criteria are not applicable to a particular agent.

The MIC_90_ values for aztreonam–nacubactam, cefepime–nacubactam and nacubactam were 2, 4 and >256 mg/L, respectively, when tested against the 917 clinical isolates of CRE identified in the study (Table 4). Individually, aztreonam, cefepime and nacubactam were inactive (MIC_90_, ≥64 mg/L) against CRE. In combination, aztreonam–nacubactam and cefepime–nacubactam in a 1:1 ratio demonstrated a ≥ 16-fold reduction in MIC_90_ compared to either aztreonam, cefepime, or nacubactam alone. Comparator agent percent susceptible values for CRE were 97.7% for aztreonam–avibactam, 78.8% (EUCAST) and 94.1% (CLSI) for cefiderocol, 60.0% for ceftazidime–avibactam, 46.0% (CLSI) and 51.1% (EUCAST) for meropenem–vaborbactam, 30.4% (CLSI) and 40.7% (EUCAST) for imipenem–relebactam and 66.7% (EUCAST) for colistin.

**Table 4.** *In vitro* activity of aztreonam–nacubactam, cefepime–nacubactam and comparator agents against 917 clinical isolates of CRE^a^

				MIC interpretation^b^					
	mg/L			CLSI				EUCAST	
Antibacterial agent	MIC range	MIC_50_	MIC_90_	% S	% SDD	% I	% R	% S	% R
Aztreonam	≤0.03–>64	>64	>64	21.2	NA	2.4	76.4	17.8	78.8
Aztreonam–avibactam	≤0.015–>64	0.25	0.5	97.7	NA	1.1	1.2	97.7	2.3
Aztreonam–nacubactam	≤0.03–>64	1	2	NA	NA	NA	NA	NA	NA
Cefepime	0.015–>64	>64	>64	15.2	5.1	NA	79.7	14.4	82.9
Cefepime–nacubactam	0.015–>64	2	4	NA	NA	NA	NA	NA	NA
Cefiderocol	≤0.03–>32	1	4	94.1	NA	3.1	2.8	78.8	21.2
Ceftazidime	≤0.03–>32	>32	>32	16.2	NA	0.9	82.9	13.8	83.8
Ceftazidime–avibactam	≤0.015–>64	2	>64	60.0	NA	0	40.0	60.0	40.0
Colistin	≤0.12–>16	0.25	>16	0	NA	66.7	33.3	66.7	33.3
Imipenem	0.12–>32	16	>32	1.5	NA	2.3	96.2	3.8	72.2
Imipenem–relebactam	0.06–>32	4	>32	30.4	NA	10.3	59.3	40.7	59.3
Meropenem	0.008–>16	>16	>16	17.4	NA	2.2	80.4	19.6	66.4
Meropenem–vaborbactam	0.008–>16	8	>16	46.0	NA	5.1	48.9	51.1	48.9
Nacubactam	1–>256	2	>256	NA	NA	NA	NA	NA	NA
Piperacillin–tazobactam	≤0.25–>32	>32	>32	15.0	1.0	NA	84.0	15.0	85.0

^a^CRE were defined as isolates of *Enterobacterales* with an imipenem MIC of ≥4 mg/L and/or a meropenem MIC of ≥4 mg/L.

^b^S, susceptible; SDD, susceptible-dose dependent; I, intermediate; R, resistant; NA, there are no MIC breakpoints available for this agent, or there are no MIC breakpoint criteria for this interpretative category, or the MIC breakpoint criteria are not applicable to a particular agent.

Table 5 and Table 6 summarize the *in vitro* activity of aztreonam–nacubactam and cefepime–nacubactam against clinical isolates of *Enterobacterales* resistant to carbapenems, newer β-lactam/β-lactamase inhibitor combinations, cefiderocol and colistin. The MIC_90_ values for aztreonam–nacubactam ranged from 2 to 8 mg/L, and for cefepime–nacubactam from 4 to 8, for carbapenem-resistant, ceftazidime–avibactam-resistant, imipenem–relebactam-resistant, meropenem–vaborbactam-resistant, aztreonam–avibactam-resistant and cefiderocol-resistant isolates.

**Table 5.** MIC frequency distributions for aztreonam–nacubactam tested against all isolates of *Enterobacterales* and against carbapenem-resistant, ceftazidime–avibactam-resistant, imipenem–relebactam-resistant, meropenem–vaborbactam-resistant, aztreonam–avibactam-resistant, cefiderocol-resistant and colistin-resistant isolates

Phenotype^a^	*n*	MIC, mg/L Number of isolates with aztreonam–nacubactam MIC Cumulative percentage of isolates inhibited at MIC													mg/L	
		≤0.03	0.06	0.12	0.25	0.5	1	2	4	8	16	32	64	>64	MIC_50_	MIC_90_
All Enterobacterales	12 613	2900	3940	1943	1007	1279	1077	398	50	6	2	2	1	8	0.06	1
		23.0	54.2	69.6	77.6	87.8	96.3	99.5	99.8	99.9	99.9	99.9	99.9	100		
Carbapenem-resistant	917	96	29	20	46	65	336	288	25	4		1	1	6	1	2
		10.5	13.6	15.8	20.8	27.9	64.6	96.0	98.7	99.1		99.2	99.3	100		
Ceftazidime–avibactam-resistant	382	5	11	11	25	51	171	93	8	3	1			3	1	2
		1.3	4.2	7.1	13.6	27.0	71.7	96.1	98.2	99.0	99.2			100		
Imipenem–relebactam-resistant^b^	452	2	8	7	25	49	199	144	12	1		1	1	3	1	2
		0.4	2.2	3.8	9.3	20.1	64.2	96.0	98.7	98.9		99.1	99.3	100		
Meropenem–vaborbactam-resistant	448	3	6	4	15	39	210	159	10	1				1	1	2
		0.7	2.0	2.9	6.3	15.0	61.8	97.3	99.6	99.8				100		
Aztreonam–avibactam-resistant	41		1				7	22	5	3	1			2	2	8
			2.4				19.5	73.2	85.4	92.7	95.1			100		
Cefiderocol-resistant	41				1	8	19	11	1					1	1	2
					2.4	22.0	68.3	95.1	97.6					100		
Colistin-resistant	2275	1106	333	374	136	94	131	83	10	2	1	1		4	0.06	1
		48.6	63.3	79.7	85.7	89.8	95.6	99.2	99.6	99.7	99.8	99.8		100		

^a^Carbapenem-resistant, ≥4 mg/L for imipenem and/or ≥4 mg/L for meropenem; ceftazidime–avibactam-resistant, ≥16 mg/L; imipenem–relebactam-resistant, ≥4 mg/L; meropenem–vaborbactam-resistant, ≥16 mg/L; aztreonam–avibactam-resistant, ≥8 mg/L (EUCAST-resistant MIC breakpoint; CLSI not susceptible MIC breakpoint); cefiderocol-resistant, ≥16 mg/L (CLSI resistant MIC breakpoint); colistin-resistant, ≥4 mg/L.

^b^Isolates of *Morganellaceae* were excluded from this resistant subset because imipenem–relebactam MIC breakpoints do not exist for this group of organisms.

**Table 6.** MIC frequency distributions for cefepime-nacubactam against all isolates of *Enterobacterales* and against carbapenem-resistant, ceftazidime–avibactam-resistant, imipenem–relebactam-resistant, meropenem–vaborbactam-resistant, aztreonam–avibactam-resistant, cefiderocol-resistant and colistin-resistant isolates

Phenotype^a^	*n*	MIC, mg/L Number of isolates with cefepime–nacubactam MIC Cumulative percentage of isolates inhibited at MIC															mg/L	
		≤0.008	0.015	0.03	0.06	0.12	0.25	0.5	1	2	4	8	16	32	64	>64	MIC_50_	MIC_90_
All Enterobacterales	12 613	43	434	4300	3508	1180	1217	915	485	405	70	27	7	12	7	3	0.06	0.5
		0.3	3.8	37.9	65.7	75.0	84.7	91.9	95.8	99.0	99.6	99.8	99.8	99.9	100	100		
Carbapenem-resistant	917		5	53	48	12	15	73	248	343	68	25	7	11	6	3	2	4
			0.5	6.3	11.6	12.9	14.5	22.5	49.5	86.9	94.3	97.1	97.8	99.0	99.7	100		
Ceftazidime–avibactam-resistant	382			1	2				75	197	57	26	7	7	7	3	2	8
				0.3	0.8				20.4	72.0	86.9	93.7	95.5	97.4	99.2	100		
Imipenem–relebactam-resistant^b^	452			1		1	2	5	112	243	51	20	6	8	3		2	4
				0.2		0.4	0.9	2.0	26.8	80.5	91.8	96.2	97.6	99.3	100			
Meropenem–vaborbactam-resistant	448			1	1			1	114	237	58	16	6	5	6	3	2	4
				0.2	0.4			0.7	26.1	79.0	92.0	95.5	96.9	98.0	99.3	100		
Aztreonam–avibactam-resistant	41								14	16	4	3		2	2		2	8
									34.1	73.2	82.9	90.2		95.1	100			
Cefiderocol-resistant	41						2	6	12	13	6			2			2	4
							4.9	19.5	48.8	80.5	95.1			100				
Colistin-resistant	2275	3	67	418	1019	325	140	96	75	82	24	10	5	4	4	3	0.06	0.5
		0.1	3.1	21.5	66.2	80.5	86.7	90.9	94.2	97.8	98.9	99.3	99.5	99.7	99.9	100		

^a^Carbapenem-resistant, ≥4 mg/L for imipenem and/or ≥4 mg/L for meropenem; ceftazidime–avibactam-resistant, ≥16 mg/L; imipenem–relebactam-resistant, ≥4 mg/L; meropenem–vaborbactam-resistant, ≥16 mg/L; aztreonam–avibactam-resistant, ≥8 mg/L(EUCAST resistant MIC breakpoint; CLSI not susceptible MIC breakpoint); cefiderocol-resistant, ≥16 mg/L (CLSI resistant MIC breakpoint); colistin-resistant, ≥4 mg/L.

^b^Isolates of *Morganellaceae* were excluded from this resistant subset because imipenem–relebactam MIC breakpoints do not exist for this group of organisms.

## Discussion

In the current study, we determined MICs for over 12 000 recent clinical isolates of *Enterobacterales* against two investigational agents (aztreonam–nacubactam, cefepime–nacubactam) with activity against CRE that have recently completed Phase III trials (regulatory filing in preparation).^10^ We observed that both aztreonam–nacubactam (1:1 ratio) and cefepime–nacubactam (1:1 ratio) inhibited 99.8% of all *Enterobacterales* isolates tested at MICs of 4 mg/L (corresponding to the CLSI susceptible breakpoint for aztreonam monotherapy) and 8 mg/L (corresponding to the CLSI susceptible dose-dependent breakpoint for cefepime monotherapy).^14^ Our study represents the largest collection of clinical isolates tested against these two investigational compounds to date.

Previously, Mushtaq *et al*. reported that cefepime (8 mg/L) combined with nacubactam (4 mg/L) inhibited 278 of 309 (90.0%) of *Enterobacterales* isolates tested while aztreonam (1 mg/L) combined with nacubactam (4 mg/L) inhibited 305 of 309 (98.7%) of isolates.^12^ In the same report, Mushtaq *et al*., testing nacubactam at a fixed concentration of 4 mg/L, reported that cefepime–nacubactam was highly active against consecutive and supplemental clinical isolate collections of MBL-producing Enterobacterales (158 NDM, 52 VIM, 99 IMP), regardless of both MBL type and aztreonam-resistance status.^12^ For nacubactam alone, Mushtaq *et al*., reported values for Proteeae that were almost all >32 mg/L, whereas those for the other *Enterobacterales* genera tested (*E. coli*, *Enterobacter* spp., *Citrobacter* spp., *Klebsiella* spp.) were bimodal, with a large peak at 1–8 mg/L and a much smaller peak at >32 mg/L.^12^ In the current study, we noted that 23.0% (313/1356) of Proteeae isolates (*Proteus* spp., *Providencia* spp., *Morganella* spp.) tested with nacubactam MICs of >256 mg/L, whereas only 3.5% (399/11 257) of other species of *Enterobacterales* tested with MICs that high (data not shown). Interestingly, of the 49 Proteeae isolates identified in our study as aztreonam resistant by the CLSI breakpoint (MIC, ≥16 mg/L), 84% demonstrated a decrease in MIC to below the aztreonam susceptible breakpoint (MIC, ≤4 mg/L) with the addition of nacubactam at a 1:1 ratio. Similarly, of the 81 Proteeae isolates that were cefepime resistant by the CLSI breakpoint (MIC, ≥16 mg/L), 85% demonstrated a decrease in MIC to below the cefepime dose-dependent susceptible breakpoint (MIC, ≤8 mg/L) with the addition of nacubactam at a 1:1 ratio (data not shown). These observations suggest that, in the case of Proteeae, nacubactam’s activity is primarily due to its β-lactamase inhibitory activity and not to its PBP2-targeting antibacterial activity.

Le Terrier *et al*. evaluated aztreonam–nacubactam (nacubactam at a fixed concentration of 4 mg/L) against 64 isolates of *Enterobacterales* clinical isolates producing NDM, VIM, IMP and SPM MBLs.^17^ Aztreonam–nacubactam inhibited 84.4% of isolates.^17^ Blanco-Martin *et al*. studied 57 isolates of double-carbapenemase-producing *Enterobacterales* and found aztreonam–nacubactam MIC_50_ and MIC_90_ values of 1 and 2 mg/L (all isolates inhibited at 4 mg/L) and cefepime–nacubactam MIC_50_ and MIC_90_ values of 2 and 4 mg/L (all isolates inhibited at 16 mg/L) with nacubactam tested at a 1:1 ratio with both aztreonam and cefepime.^11^ Aztreonam–nacubactam tested at a fixed concentration of nacubactam of 4 mg/L generated MIC_50_ and MIC_90_ values of ≤0.25 and ≤0.25 mg/L, respectively, which were similar to aztreonamIMPavibactam (MIC_50_ and MIC_90_ values of ≤0.25 and 0.5 mg/L) where avibactam is tested at a fixed concentration of 4 mg/L.^11,14^ Livermore *et al*. reported porin loss may restrict entry of nacubactam into *E. coli*, *Klebsiella* spp. and *Enterobacter* spp.^8^ Specific mutations in *ileS* in *E. coli*, which encodes aminoacyl tRNA synthesis and modification function, may be a potential contributor to nacubactam resistance, although the frequency and importance of this mechanism to the activities of combination agents containing nacubactam remains to be determined.^13,18^ Liu *et al*. tested 204 NDM-producing *Enterobacterales* collected in China against aztreonam–nacubactam and aztreonam–avibactam and reported that nacubactam reduced the MIC_50_ and MIC_90_ values for aztreonam from 256 and >256 to 1 and 2 mg/L, respectively, while avibactam reduced the MIC_50_ and MIC_90_ values for aztreonam from 256 and >256 to 0.12 and 1 mg/L, respectively.^13^

Combining each of aztreonam and cefepime with nacubactam is rational for several reasons. Aztreonam is a monobactam that resists degradation by MBLs and inhibits bacterial cell wall synthesis primarily by targeting PBP3. Cefepime is a broad-spectrum cephem that binds primarily not only to PBP3 but also to PBP1a in *Enterobacterales* and retains activity against AmpC derepressed species of *Enterobacterales* because AmpC has a low affinity for cefepime. When aztreonam or cefepime is combined with nacubactam, the concomitant inactivation of multiple PBPs leads to enhanced antibacterial activity. In addition, even though nacubactam does not directly inhibit MBLs or class D carbapenemases, it can enhance the activities of aztreonam and cefepime against isolates carrying these enzymes via its PBP2 binding. Nacubactam is also highly stable to degradation by both serine β-lactamases and MBLs, a feature distinguishing it from DBOs currently marketed in other combinations (aztreonam–avibactam, ceftazidime–avibactam, imipenem–relebactam).^8,9,12,13,17,19^ Both aztreonam and cefepime have multiple clinical indications in their current FDA product package inserts.^20,21^ Combining aztreonam or cefepime with nacubactam offers a potential treatment for infections with a current aztreonam or cefepime indication caused by isolates of *Enterobacterales* resistant to aztreonam or cefepime alone, including both KPC and MBL-producing CRE isolates.

In conclusion, we studied a recent worldwide collection of 12 613 *Enterobacterales* and observed that aztreonam–nacubactam (MIC_90_, 1 mg/L) and cefepime–nacubactam (MIC_90_, 0.5 mg/L) demonstrated potent *in vitro* activity against *Enterobacterales*, including CRE (aztreonam–nacubactam MIC_90_, 2 mg/L; cefepime–nacubactam MIC_90_, 4 mg/L) for which treatment options are currently limited.

## Supplementary Material

dlag061_Supplementary_Data

## Supplementary data

Tables S1 to S4 are available as Supplementary data at *JAC-AMR* Online.
