# Supplementary material for: In vitro activity of aztreonam–nacubactam and cefepime–nacubactam against a global collection of clinically important Gram-negative Bacilli collected in 2021–2023
Source: JAC Antimicrob Resist. 2026 May 5;8(3):dlag061. doi: 10.1093/jacamr/dlag061 (PMC13143013; doi:10.1093/jacamr/dlag061)
Supplement: dlag061_Supplementary_Data [file dlag061_supplementary_data.docx]

**Supplemental Table 1**

Geographical summary of the 16,962 Gram-negative bacilli isolates tested in the study

| Geographical region | Country | No. of laboratory sites | No. (%) of *Enterobacterales* isolates | No. (%) of *Pseudomonas aeruginosa* isolates | No. (%) of *Acinetobacter baumannii* isolates |
| --- | --- | --- | --- | --- | --- |
| Asia | India | 3 | 357 (2.8) | 90 (2.4) | 30 (5.0) |
|  | Hong Kong | 3 | 359 (2.8) | 109 (2.9) | 23 (3.8) |
|  | Japan | 4 | 867 (6.9) | 234 (6.2) | 17 (2.8) |
|  | Malaysia | 4 | 623 (4.9) | 187 (5.0) | 43 (7.2) |
|  | South Korea | 3 | 421 (3.3) | 116 (3.1) | 30 (5.0) |
|  | Taiwan | 5 | 516 (4.1) | 176 (4.7) | 38 (6.3) |
|  | Thailand | 5 | 799 (6.3) | 250 (6.7) | 60 (10.0) |
|  | Vietnam | 2 | 177 (1.4) | 64 (1.7) | 15 (2.5) |
| Asia total | 8 countries | 29 | 4,119 (32.7) | 1,226 (32.7) | 256 (42.6) |
|  |  |  |  |  |  |
| Europe | Belgium | 2 | 292 (2.3) | 88 (2.3) | 3 (0.5) |
|  | Bulgaria | 1 | 72 (0.6) | 22 (0.6) | 5 (0.8) |
|  | Croatia | 2 | 214 (1.7) | 66 (1.8) | 15 (2.5) |
|  | Czech Republic | 1 | 73 (0.6) | 22 (0.6) | 2 (0.3) |
|  | France | 4 | 570 (4.5) | 146 (3.9) | 12 (2.0) |
|  | Germany | 3 | 512 (4.1) | 154 (4.1) | 13 (2.2) |
|  | Greece | 1 | 216 (1.7) | 66 (1.8) | 15 (2.5) |
|  | Hungary | 2 | 291 (2.3) | 88 (2.3) | 20 (3.3) |
|  | Italy | 4 | 705 (5.6) | 219 (5.8) | 39 (6.5) |
|  | Latvia | 1 | 146 (1.2) | 44 (1.2) | 10 (1.7) |
|  | Lithuania | 1 | 219 (1.7) | 66 (1.8) | 15 (2.5) |
|  | Norway | 1 | 66 (0.5) | 19 (0.5) | 1 (0.2) |
|  | Poland | 2 | 292 (2.3) | 79 (2.1) | 20 (3.3) |
|  | Portugal | 3 | 274 (2.2) | 64 (1.7) | 4 (0.7) |
|  | Romania | 1 | 213 (1.7) | 61 (1.6) | 15 (2.5) |
|  | Serbia | 1 | 137 (1.1) | 44 (1.2) | 10 (1.7) |
|  | Spain | 6 | 870 (6.9) | 264 (7.0) | 9 (1.5) |
|  | Sweden | 1 | 72 (0.6) | 22 (0.6) | 1 (0.2) |
|  | Switzerland | 1 | 218 (1.7) | 66 (1.8) | 2 (0.3) |
|  | Turkey | 4 | 392 (3.1) | 126 (3.4) | 30 (5.0) |
|  | Ukraine | 1 | 210 (1.7) | 66 (1.8) | 15 (2.5) |
|  | United Kingdom | 3 | 434 (3.4) | 132 (3.5) | 9 (1.5) |
| Europe total | 22 countries | 46 | 6,488 (51.4) | 1,924 (51.3) | 265 (44.1) |
|  |  |  |  |  |  |
| North America | United States | 14 | 1,730 (13.7) | 510 (13.6) | 77 (12.8) |
| North America total | 1 country | 14 | 1,730 (13.7) | 510 (13.6) | 77 (12.8) |
|  |  |  |  |  |  |
| South Pacific | Australia | 3 | 204 (1.6) | 66 (1.8) | 2 (0.3) |
|  | New Zealand | 1 | 72 (0.6) | 22 (0.6) | 1 (0.2) |
| South Pacific total | 2 countries | 4 | 276 (2.2) | 88 (2.3) | 3 (0.5) |
|  |  |  |  |  |  |
| Grand total | 33 countries | 93 sites | 12,613 (100) | 3,748 (100) | 601 (100) |

**Supplemental Table 2**

Infection source and year of collection for the 16,962 Gram-negative bacilli isolates tested in the study

|  | Isolate infection source  No. of isolates tested | | | | | |  | Year of isolate collection  No. of isolates tested | | |
| --- | --- | --- | --- | --- | --- | --- | --- | --- | --- | --- |
| Order/genus and species | Respiratory | Genitourinary | Blood | Gastrointestinal | Sterile fluid | Unknown |  | 2021 | 2022 | 2023 |
| *Enterobacterales* | 4,768 | 2,977 | 2,359 | 1,236 | 1,210 | 63 |  | 3,656 | 3,695 | 5,262 |
| *Pseudomonas aeruginosa* | 2,376 | 494 | 356 | 230 | 275 | 17 |  | 1,114 | 1,083 | 1,551 |
| *Acinetobacter baumannii* | 412 | 49 | 91 | 17 | 29 | 3 |  | 196 | 188 | 217 |

**Supplemental Table 3**

*In vitro* activity of aztreonam-nacubactam, cefepime-nacubactam, and comparator agents against 3,748 clinical isolates of *Pseudomonas aeruginosa*

| Antibacterial agent |  | | |  | MIC interpretation^a^ | | | | | |
| --- | --- | --- | --- | --- | --- | --- | --- | --- | --- | --- |
|  | µg/mL | | |  | CLSI | | |  | EUCAST | |
|  | MIC range | MIC_50_ | MIC_90_ |  | % S | % I | % R |  | % S | % R |
| Aztreonam | ≤0.03–>64 | 8 | 32 |  | 70.9 | 10.5 | 18.6 |  | 0 | 18.6 |
| Aztreonam-avibactam | ≤0.015–>64 | 4 | 32 |  | NA | NA | NA |  | NA | NA |
| Aztreonam-nacubactam | ≤0.03–>64 | 4 | 16 |  | NA | NA | NA |  | NA | NA |
| Cefepime | 0.03–>64 | 2 | 32 |  | 82.3 | 6.9 | 10.8 |  | 0 | 17.7 |
| Cefepime-nacubactam | 0.03–>64 | 2 | 8 |  | NA | NA | NA |  | NA | NA |
| Cefiderocol | ≤0.03–>32 | 0.25 | 1 |  | 99.0 | 0.4 | 0.6 |  | 96.8 | 3.2 |
| Ceftazidime | 0.06–>32 | 2 | >32 |  | 78.7 | 3.6 | 17.7 |  | 0 | 21.3 |
| Ceftazidime-avibactam | ≤0.015–>64 | 2 | 8 |  | 91.4 | 0 | 8.6 |  | 91.4 | 8.6 |
| Colistin | ≤0.12–>16 | 0.5 | 1 |  | 0 | 99.5 | 0.5 |  | 99.7 | 0.3 |
| Imipenem | ≤0.03–>32 | 1 | 16 |  | 71.9 | 4.7 | 23.4 |  | 0 | 23.4 |
| Imipenem-relebactam | ≤0.03–>32 | 0.5 | 2 |  | 90.5 | 3.4 | 6.1 |  | 90.5 | 9.5 |
| Meropenem | ≤0.004–>16 | 0.5 | 16 |  | 76.7 | 5.4 | 17.9 |  | 76.7 | 11.9 |
| Meropenem-vaborbactam | ≤0.004–>16 | 0.5 | 16 |  | NA | NA | NA |  | 88.5 | 11.5 |
| Nacubactam | 1–>256 | 128 | 128 |  | NA | NA | NA |  | NA | NA |
| Piperacillin-tazobactam | ≤0.25–>32 | 8 | >32 |  | 75.5 | 6.9 | 17.7 |  | 0 | 24.5 |

^a^ S, susceptible; I, intermediate; R, resistant; NA, there are no MIC breakpoints available for this agent, or there are no MIC breakpoint criteria for this interpretative category, or the MIC breakpoint criteria are not applicable to a particular agent.

**Supplemental Table 4**

*In vitro* activity of aztreonam-nacubactam, cefepime-nacubactam, and comparator agents against 601 clinical isolates of *Acinetobacter baumannii*

| Antibacterial agent | MIC | | |  | MIC interpretation^a^ | | | | | |
| --- | --- | --- | --- | --- | --- | --- | --- | --- | --- | --- |
|  | µg/mL | | |  | CLSI | | |  | EUCAST | |
|  | MIC range | MIC_50_ | MIC_90_ |  | % S | % I | % R |  | % S | % R |
| Aztreonam | 2–>64 | 64 | >64 |  | NA | NA | NA |  | NA | NA |
| Aztreonam-avibactam | ≤0.015–>64 | 64 | >64 |  | NA | NA | NA |  | NA | NA |
| Aztreonam-nacubactam | 0.5–>64 | 32 | >64 |  | NA | NA | NA |  | NA | NA |
| Cefepime | 0.25–>64 | 32 | >64 |  | 29.3 | 6.3 | 64.4 |  | NA | NA |
| Cefepime-nacubactam | 0.25–64 | 16 | 64 |  | NA | NA | NA |  | NA | NA |
| Cefiderocol | ≤0.03–>32 | 0.5 | 4 |  | 91.3 | 1.2 | 7.5 |  | NA | NA |
| Ceftazidime | 0.06–>32 | >32 | >32 |  | 30.9 | 0.8 | 68.2 |  | NA | NA |
| Ceftazidime-avibactam | 0.06–>64 | 32 | >64 |  | NA | NA | NA |  | NA | NA |
| Colistin | ≤0.12–16 | 0.25 | 0.5 |  | 0 | 98.3 | 1.7 |  | 98.3 | 1.7 |
| Imipenem | ≤0.03–>32 | 32 | >32 |  | 29.1 | 0.8 | 70.0 |  | 29.1 | 70.0 |
| Imipenem-relebactam | ≤0.03–>32 | 32 | >32 |  | 30.6 | 1.0 | 68.4 |  | NA | NA |
| Meropenem | 0.03–>16 | >16 | >16 |  | 29.5 | 0.5 | 70.0 |  | 29.5 | 68.9 |
| Meropenem-vaborbactam | 0.015–>16 | >16 | >16 |  | NA | NA | NA |  | NA | NA |
| Nacubactam | 2–>256 | >256 | >256 |  | NA | NA | NA |  | NA | NA |
| Piperacillin-tazobactam | ≤0.25–>32 | >32 | >32 |  | 28.0 | 72.0 | 0 |  | NA | NA |

^a^ S, susceptible; I, intermediate; R, resistant; NA, there are no MIC breakpoints available for this agent, or there are no MIC breakpoint criteria for this interpretative category, or the MIC breakpoint criteria are not applicable to a particular agent.
